# Aspergillus Fumigatus Spore Proteases Alter the Respiratory Mucosa Architecture and Facilitate Equine Herpesvirus 1 Infection

**DOI:** 10.3390/v16081208

**Published:** 2024-07-27

**Authors:** Joren Portaels, Eline Van Crombrugge, Wim Van Den Broeck, Katrien Lagrou, Kathlyn Laval, Hans Nauwynck

**Affiliations:** 1 Department of Translational Physiology, Infectiology and Public Health, Faculty of Veterinary Medicine, Ghent University, 9820 Merelbeke, Belgium; joren.portaels@ugent.be (J.P.); esvcromb.vancrombrugge@ugent.be (E.V.C.); 2 Department of Morphology, Medical Imaging, Orthopedics and Nutrition, Faculty of Veterinary Medicine, Ghent University, 9820 Merelbeke, Belgium; wim.vandenbroeck@ugent.be; 3 Department of Microbiology, Immunology and Transplantation, Laboratory of Clinical Microbiology, 3000 Leuven, Belgium; katrien.lagrou@uzleuven.be

**Keywords:** aspergillus fumigatus, fungal spores, EHV-1, proteases, cell junctions, equine respiratory mucosal explants, mucosal barriers

## Abstract

Numerous *Aspergillus fumigatus* (Af) airborne spores are inhaled daily by humans and animals due to their ubiquitous presence. The interaction between the spores and the respiratory epithelium, as well as its impact on the epithelial barrier function, remains largely unknown. The epithelial barrier protects the respiratory epithelium against viral infections. However, it can be compromised by environmental contaminants such as pollen, thereby increasing susceptibility to respiratory viral infections, including alphaherpesvirus equine herpesvirus type 1 (EHV-1). To determine whether Af spores disrupt the epithelial integrity and enhance susceptibility to viral infections, equine respiratory mucosal ex vivo explants were pretreated with Af spore diffusate, followed by EHV-1 inoculation. Spore proteases were characterized by zymography and identified using mass spectrometry-based proteomics. Proteases of the serine protease, metalloprotease, and aspartic protease groups were identified. Morphological analysis of hematoxylin-eosin (HE)-stained sections of the explants revealed that Af spores induced the desquamation of epithelial cells and a significant increase in intercellular space at high and low concentrations, respectively. The increase in intercellular space in the epithelium caused by Af spore proteases correlated with an increase in EHV-1 infection. Together, our findings demonstrate that Af spore proteases disrupt epithelial integrity, potentially leading to increased viral infection of the respiratory epithelium.

## 1. Introduction

*Aspergillus fumigatus* (Af) is a saprophytic fungus that is ubiquitous in soil and grows in places with plant debris. This fungus is, thus, frequently found in compost heaps, straw bales, or stacked hay, resulting in many spores being produced. Growth and spore production occur throughout the year with great extent at autumn and winter [1]. Potential sources in enclosed spaces include poorly functioning or contaminated ventilation, moist wood, potting soil, and construction work [2]. An important reservoir of *Aspergillus fumigatus* spores is dust accumulation, frequently found in attics, basements, and uncleaned spaces where the spores survive without developing fungal mycelium. Environmental concentrations of Af spores are estimated to be between 1 and 100 conidia/m^3^. Consequently, an average adult may inhale more than 100 conidia each day [3]. Conidia have a small size of 2–3 µm and are hydrophobic, which allows them to be easily remain airborne for long periods and to easily reach the lung alveoli after inhalation [3,4,5].

Af is included in the WHO’s first fungal priority pathogens list as it ranked the highest for perceived public health importance [6]. It is a pathogen that can cause a large variety of diseases in humans and animals [7]. Exposure to allergens of the conidia of Af triggers an IgE-mediated allergic immune response leading to bronchial obstruction and asthma, which can progress to severe asthma with fungal sensitization (SAFS) or allergic bronchopulmonary aspergillosis (ABPA) [8,9]. Non-invasive saprophytic growth can cause a mycotic rhinitis and sinusitis with mycotic plaques and exudate that contains fungal hyphae and spores [10,11]. In horses, guttural pouch mycosis is a rare but life-threatening result of saprophytic growth of mainly aspergillus sp., including Af, in the guttural pouch that extends from the pharynx [12,13]. When reaching the lungs, the saprophytic growth is observed as a pulmonary aspergilloma or “fungus ball” which can be life-threatening because of hemoptysis [7,14]. Invasive aspergillosis is mainly seen in immunocompromised patients and presents as an acute or chronic pulmonary aspergillosis, an invasive rhinosinusitis, a tracheobronchitis with invasion into the respiratory mucosa and the tracheobronchial cartilage, or a disseminated form of aspergillosis with spread to the brain and other organs such as the kidneys, heart, eyes, and skin [15]. 

Inhaled Af conidia are cleared by a healthy host’s innate immune defense including mucociliary transport, antifungal antimicrobial peptides, and phagocytosis [16,17]. It has been demonstrated that Af conidia are able to bind and be internalized by tracheal epithelial cells [18]. The internalization of conidia is followed by phagolysosome acidification, an additional defense mechanism [19]. It was proposed that the fungus facilitates colonization by secreting proteases that alter epithelial function via cell shrinkage, desquamation, and actine cytoskeleton rearrangements [20,21,22]. The conidial wall has been shown to have proteolytic activity with an aspartic protease (Pep2) and lipase activity with an extracellular lipase [23]. Recently, the conidia-associated metalloprotease Mep1p was found to facilitate early immune evasion [24]. Still, the interaction of Af conidia with the respiratory epithelia remains to be further investigated. 

The alphaherpesvirus equine herpesvirus type 1 (EHV-1) is a highly prevalent pathogen that has a significant impact on the horse industry worldwide [25,26]. EHV-1 can cause respiratory disorders, abortion, neonatal foal death, and EHV-1 myeloencephalopathy in horses [27,28]. The respiratory epithelium of the upper respiratory tract is the main entry point of EHV-1. The virus spreads in a plaque-like manner over the epithelium of the nasal septum, nasopharynx, and trachea [29]. Then, EHV-1 crosses the basement membrane via infected mononuclear immune cells to reach the blood vessels of the lamina propria. The virus causes a cell-associated viremia, primarily targeting monocytes and T-lymphocytes. Subsequently, EHV-1 further disseminates to target organs such as the central nervous system or the uterus of gestating horses [29,30,31]. A secondary replication occurs in the endothelial cells lining the blood vessels of these organs and can cause vasculitis and ischemic thrombosis, potentially leading to abortion and/or neurological disorders [27,32,33,34]. In the lamina propria, the virus can also infect sensory nerve endings after which the virus can travel towards the trigeminal ganglion to establish latency [35].

The respiratory epithelium has different innate mucosal barriers, including a mucus layer with the mucociliary transport, the intercellular connections, and the production of antimicrobial peptides [36]. It has previously been shown that the disruption of intercellular junctions (ICJ) greatly increases the susceptibility to EHV-1 binding. The researchers, therefore, proposed that the compromised integrity of the respiratory epithelium may facilitate EHV1 infection by exposing basolateral entry receptors present on epithelial cells. To date, the basolateral receptor for EHV-1 has not been characterized yet [37]. Similarly, it was demonstrated that the mycotoxin deoxynivalenol and the exotoxins α-hemolysin from *Staphylococcus aureus* and adenylate cyclase toxin from *Bordetella bronchiseptica*, can increase the horse’s respiratory mucosa’s susceptibility to EHV-1 infection by compromising the respiratory epithelial barrier [38,39]. Moreover, pollen proteases also disrupt ICJ and the anchorage of respiratory epithelial cells in both ex vivo equine respiratory mucosal explants and in vitro primary equine respiratory epithelial cells [40].

So far, it is not known whether the Af spores can also predispose the horse’s respiratory epithelium to EHV-1 infection. Therefore, this study demonstrates the effects of ubiquitous Af spores on the respiratory epithelium and its susceptibility to EHV-1 infection, using an in-house-developed ex vivo horse trachea explant system.

## 2. Materials and Methods

### 2.1. Spore Diffusate Collection

The spore diffusate of the fungus *Aspergillus fumigatus* was collected after 5 days of cultivation of both a wild type (WT) strain and a clinical isolate (3414) using 0.01% Tween20^®^ (Sigma-Aldrich, St. Louis, MO, USA) in Dulbecco’s Phosphate-Buffered saline (DPBS) (ThermoFisher Scientific, Waltham, MA, USA), as described by Van Waeyenberghe et al. [41]. The spore diffusate was then filtered through a 20 μm membrane filter (Millipore^®^, Sigma-Aldrich). Immediately before use, the diffusate was filtered a second time with a 30 kilodalton (kDa) molecular weight cutoff filter (Millipore^®^, Sigma-Aldrich). The protein concentration was determined using a PierceTM BCA Protein Assay Kit (ThermoFisher Scientific) and a Nanodrop^®^ spectrophotometer. The protein concentration in WT and clinical spore diffusates was 0.518 mg/mL and 2.628 mg/mL, respectively. 

### 2.2. Gel Electrophoresis and Zymography

To determine the protein and proteolytic profiles of the spore diffusate, SDS-polyacrylamide gel electrophoresis (SDS-PAGE) and zymography were performed. The gel electrophoresis was performed by loading the spore diffusates, diluted 1:1 in Laemmli sample buffer, onto a 10% SDS-polyacrylamide gel. Additionally, a protein ladder (PageRuler Plus Prestained Protein Ladder, ThermoFisher Scientific) and bovine plasmin (500 µg/mL; Innovative Research, Novi, MI, USA) (1:1 diluted in Laemmli buffer) were loaded as markers for the proteins and for the proteolytic activities. The gel was run at 150 volts for 2 h until the 28 kDa marker on the ladder was passed. After washing the gel 3 times for 5 min with ultra-pure (UP) water, the gel was stained for 1 h with Coomassie blue R-250 (Imperial™ Protein Stain; ThermoFisher Scientific) and destained for 2 h in UP water. The gel was kept in UP water overnight. The zymography was performed on a 10% SDS-polyacrylamide gel containing gelatine (4 mg/mL, Sigma-Aldrich). After running the gel in parallel with the SDS-PAGE, it was washed twice for 30 min with washing buffer (2.5% Triton X-100, 50 mM Tris-HCl, pH 7.5, 5 mM CaCl_2_, and 1 μM ZnCl_2_) to renature the proteins. The gel was further incubated for 12 h in buffer (1% Triton X-100, 50 mM Tris-HCl, pH 7.5, 5 mM CaCl_2_, and 1 μM ZnCl_2_) at 37 °C to perform protease activity. Afterwards, the gel was stained for 30 min with 100 mL staining solution (40 mL methanol, 10 mL acetic acid, 50 mL UP, and 0.5 g Coomassie Blue) and destained 3 times for 5 min with a solution containing 200 mL methanol, 50 mL acetic acid, and 250 mL UP. All these steps were performed on a platform shaker (Polymax 1040, Heidolph, Schwbach, Germany) for both gels. Pictures of both gels were made with ChemiDocMP Imaging System (Bio-Rad Laboratories, Hercules, CA, USA). Using ImageJ (version 1.54f), the pictures of the zymogram were converted to 8-bit gray-scaled images whereafter the proteolytic profile was determined by plot-profiling.

### 2.3. Proteomics

Three large regions of the SDS-PAGE with multiple bands (A–C) were excised in a laminar flow cabinet. The excised gel bands were washed with 500 µL UP water and stored at −20 °C. The bands were sent to VIB proteomics core (VIB-Ugent center for Medical Biotechnology in Ghent, Belgium) for mass spectrometry (MS)-based proteomics. 

LC-MS/MS runs of all samples were examined separately using the MaxQuant algorithm (version 2.4.13.0) with mainly default search settings, including a false discovery rate set at 1% on PSM and protein level. Spectra were searched against the following protein sequence database: *Aspergillus fumigatus* (strain ATCC MYA-4609/CBS 101355/FGSC A1100/Af293) Uniprot reference database (www.uniprot.org (accessed on 11 April 2024)) (database release version January 2024, containing 9647 protein sequences). The mass spectrometry proteomics data have been deposited to the ProteomeXchange Consortium via the PRIDE [42] partner repository with the dataset identifier: PXD053508.

### 2.4. Isolation and Culturing of Ex Vivo Respiratory Mucosal Explants

Tracheas from healthy horses were collected at the slaughterhouse and brought to the lab to make mucosal explants as previously described by Vandekerckhove et al. [43]. The horse tracheas were transported to the laboratory in transport medium (phosphate-buffered saline (pbs) with calcium and magnesium supplemented with 100 U/mL penicillin (ThermoFisher Scientific, Paisley, UK), 0.1 mg/mL streptomycin (ThermoFisher Scientific), 0.1 mg/mL gentamicin (ThermoFisher Scientific), 0.1 mg/mL kanamycin (Merck, Darmstadt, Germany), and 0.25 µg/mL amphotericin B (ThermoFisher Scientific). The respiratory mucosa was carefully excised from the underlying cartilage at the proximal region of the horse trachea. Square explants of 0.5 cm^2^ were cultured for 24 h at air–liquid interface in serum-free medium (SFM; 50% Roswell Park Memorial Institute medium (RPMI, ThermoFisher Scientific), 50% Dulbecco’s Modified Eagle Medium (DMEM, ThermoFisher Scientific)) supplemented with 0.1 mg/mL streptomycin (ThermoFisher Scientific), 100 U/mL penicillin (ThermoFisher Scientific), 0.1 mg/mL gentamicin (ThermoFisher Scientific), and 0.25 μg/mL amphotericin B (ThermoFisher Scientific)) at 37 °C and 5% CO_2_. Serum-free medium was used to culture the explants, as a previous study had shown that the use of fetal calf serum resulted in enlarged epithelial cells, loss of cell–cell contacts, and a loose epithelium in porcine mucosa explants [44].

### 2.5. Spore Diffusate Treatment

The explants were cultured for 24 h after collection for acclimatation in SFM at 37 °C and 5% CO_2_. Before spore diffusate treatment, the beating of the cilia was checked to confirm explant viability, whereafter they were thoroughly washed to remove mucus and were embedded in agarose to mimic the in vivo conditions as published by Vaïro et al. [45]. The embedded explants were treated for 24 h with several dilutions of spore diffusate to determine the effect of spore proteases on the morphology of the respiratory epithelium, as well as on the infection and replication rate of EHV-1. Treatment of the explant with SFM was used as a negative control. The 1 h treatment with 8 mM Ca^2+^ chelating agent ethylene glycol tetra-acetic acid (EGTA; VWR International, Leuven, Belgium) was used as a positive control. EGTA disrupts ICJ, leading to enhanced EHV-1 binding [37]. To assess the effects on mucosal integrity, we used spore diffusate dilutions of 1:25 and 1:250, after which we further evaluated the impact on EHV-1 infectivity using the 1:250 dilution. The toxicity of the spore diffusate was determined for each dilution with a terminal deoxynucleotidyl transferase dUTP nick end-labelling (TUNEL) based In Situ Cell Death Detection Kit, Fluorescein (Roche Diagnostics Corporation, Basel, Switzerland), to guarantee explant viability (>90%) after spore diffusate treatment.

### 2.6. Assessment of the Intercellular Integrity

After treatment, explants were removed from the agarose and placed in 3.5% formaldehyde fixative for 24 h. Afterwards, the explants were placed in 70% ethanol until further tissue processing and embedding in paraffin. Paraffin-embedded tissue sections were stained with hematoxylin and eosin staining (HE). Light microscopy (Olympus IX50) images of the epithelium were made and assessed using ImageJ software (ImageJ, U.S. National Institutes of Health, Bethesda, MD, USA). The percentage of intercellular space was determined for each explant by measuring the blank space between the epithelial cells using ImageJ software for 3 different randomly chosen regions in the epithelium after manually determining the region of interest (ROI, the respiratory mucosa) and the threshold value. The thickness of the epithelium was also measured after treatment using ImageJ software.

### 2.7. Viral Infection Assay

The 97P70 EHV-1 strain was used for viral inoculation. This strain was isolated in Belgium from the lungs of an aborted foal in 1977 [46]. The 6th passage was used after 5 passages on embryonic equine lung (EEL) cells and 1 passage on Rabbit Kidney (RK13) cells. Treated explants were washed 3 times to remove the fungal spores and subsequently inoculated with 10^6.5^ TCID_50_ of the 97P70 EHV-1 strain for 1 h at 37 °C. Non-treated explants were included as control. After washing the viral inoculum, explants were further incubated at liquid–air interface in serum-free medium. At 24 h post-inoculation (hpi), explants were embedded in methylcellulose (Methocel^®^ MC, Sigma-Aldrich), snap frozen, and stored at −70 °C until further processing. 

### 2.8. Immunofluorescence Staining and Confocal Microscopy

Cryosections (16 µm thickness) of explants were made and loaded onto 3-aminopropyltriethoxysilane-coated (Sigma-Aldrich) glass slides. The slides were fixed in 100% methanol for 20 min at −20 °C. For immunofluorescence (IF) staining, slides were first incubated in an avidin/biotin solution (ThermoFisher Scientific) for 15 min at 37 °C to reduce non-specific binding due to endogenous biotin in tissues. To label the EHV-1 late glycoproteins and the basement membrane, slides were incubated with a horse polyclonal biotinylated anti-EHV-1 antibody (dilution 1:10) and a mouse monoclonal anti-collagen VII antibody (Sigma-Aldrich) (dilution 1:300) for 1 h at 37 °C. The polyclonal biotinylated anti-EHV-1 IgGs were obtained by the hyperimmunization of a horse [47]. Slides were washed 3 times for 5 min. Next, slides were incubated with streptavidin-FITC^®^ (ThermoFisher Scientific) (dilution 1:250) and Alexa fluor™️ 594-labelled goat anti-mouse antibodies (ThermoFisher Scientific) (dilution 1:500) for 1 h at 37 °C. The nuclei were counterstained with Hoechst 33342 (10 µg/mL; ThermoFisher Scientific) (dilution 1:100) for 10 min at 37 °C. The slides were mounted with glycerol-DABCO and analyzed by confocal microscopy (Leica TCS SP2 Laser Scanning Spectral Confocal System; Leica Microsystems). The number of viral plaques was counted for a total of 50 cryosections and the maximum plaque latitude of each plaque was measured using the Leica confocal LAS AF software (Leica Microsystems).

### 2.9. Statistical Analysis 

Statistical differences (*p* < 0.05) between the Af spore diffusate treatment or EGTA treatment and the mock (SFM) treatment were identified by one-way analysis of variances (ANOVA) followed by Tukey’s post-hoc test using GraphPad Prism version 6.0 for Mac OS (GraphPad Software, Boston, MA, USA).

## 3. Results

### 3.1. Aspergillus Fumigatus Spores Show Protease Activity

**Figure 1:**
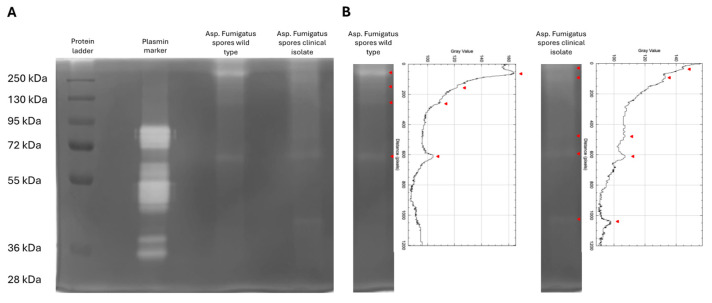
Both *Aspergillus fumigatus* strains show proteolytic activities. (**A**) The zymography with gelatin substrate was stained with Coomassie blue, whereby the proteolytic activity by proteases appears as white bands on a blue background. The zymogram shows, from left to right, a protein ladder, a plasmin marker, the Af wild type strain, and the Af clinical isolate strain. (**B**) Plot profiling of the lanes of both *Aspergillus fumigatus* strains, made in imageJ on an 8-bit grey-scaled image. Plot shows value peaks (grey) at the corresponding white proteolytic bands (white), as indicated by red arrows.

Gelatin zymography was performed to assess the proteolytic activities of spores of two Af strains. Both spore diffusates exhibited proteolytic activity with protein bands migrating in two common regions between 100 and 250 kDa and between 60 and 90 kDa. Additionally, a unique proteolytic activity band was observed for the clinical isolate in the region between 30 and 50 kDa (Figure 1A). Plot profiling of the Af lanes was performed to distinguish the presence of a white proteolytic band from the background (Figure 1B). The spore diffusate of the Af WT strain showed three proteolytic bands in the range between 100 and 250 kDa and one proteolytic band in the range between 60 and 90 kDa. The spore diffusate of the Af clinical isolate showed two proteolytic bands in the range of 100–250 kDa, two bands in the range of 60–90 kDa, and one band in the range of 30–50 kDa. Overall, we can conclude that the spore diffusate of the clinical isolate differs from the WT by an additional proteolytic band in the range of 60–90 kDa and a proteolytic band in the range of 30–50 kDa.

### 3.2. Protease Identification in the Spore Diffusate

**Figure 2:**
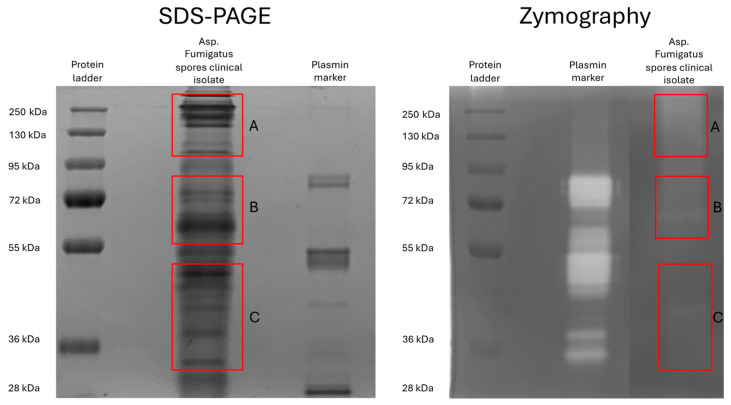
Protein (**left**) and proteolytic (**right**) profiles of the *Aspergillus fumigatus* clinical isolate spore diffusate. Three regions of the protein profile, corresponding to the three regions of proteolytic activity on the zymogram (A–C, red boxes), were excised of the SDS-PAGE and subjected to mass spectrometry (MS)-based proteomics. The zymography was adapted from Figure 1A.

Next, we identified the proteases expressed in the Af clinical isolate spores using mass spectrometry (MS)-based proteomics. As shown in Figure 2, we have cut three large bands out of the regular SDS-PAGE for LC-MS/MS analysis (red boxes with mobility at 100–250 kDa (A), 60–90 kDa (B), and 30–50 kDa (C) at corresponding enzymatic activity). The raw MS/MS data were searched against a database of *Aspergillus fumigatus* strain ATCC MYA-4609/CBS 101355/FGSC A1100/Af293, whose genome sequence, along with the annotation of its putative protein sequences, is available through the Aspergillus Genome Database (http://www.aspgd.org (accessed on 11 April 2024)) [48]. An overview of the proteases is shown in Table 1. The spores express 11 different proteases containing multiple serine proteases and metalloproteases and one aspartic protease. Besides proteases, the spores express 35 different glycosidases and a lipase, listed in the Supplementary Materials Table S1.

### 3.3. Spore Proteases Alter the Mucosal Integrity of Columnar Epithelial Cells in the Horse Respiratory Epithelium 

The treatment with spore diffusate of the Af clinical isolate 3414 does not reduce the viability of the respiratory mucosal explants (>90% living cells). After treatment, the mean percentage of living cells was 92.7 ± 2.5%, similar to the SFM treated explants, which had a mean percentage of 92.9 ± 1.1% living cells. No significant differences were found between the two treatments. Details are given in Figure S1.

**Figure 3:**
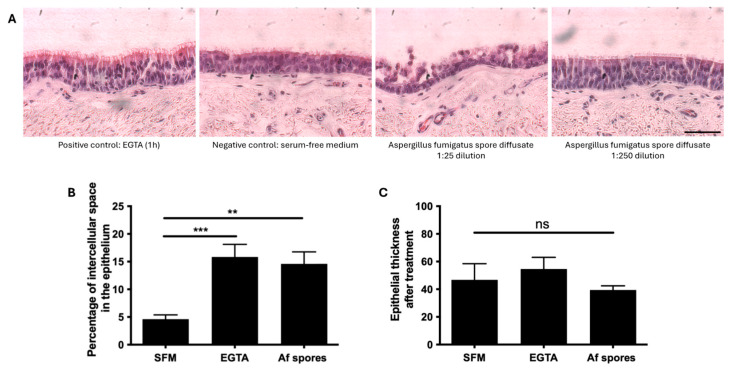
The spore diffusate alters the epithelium integrity of respiratory horse explants. (**A**) The explants were treated with EGTA for 1 h (positive control), serum-free medium (negative control), and the spore diffusate of the Af clinical isolate at a dilution of 1:25 and 1:250 for 24 h. (**B**) The percentage of intercellular space, upon Af spore diffusate treatment at a 1:250 dilution, was determined by measuring the blank space in ImageJ software after manually determining the region of interest (ROI, the respiratory epithelium) and the threshold value. (**C**) The height of the epithelium after treatment was measured simultaneously using ImageJ. Data are represented as a mean of three replicates with standard deviation. Statistical significance is indicated by asterisks (** = *p*-value < 0.01, *** = *p*-value < 0.001 and non-significance (ns)). Scale bar measures 50 µm.

To determine if the spore proteases disrupt the epithelial ICJ, ex vivo respiratory explants were treated with spore diffusate of the Af clinical isolate 3414. As seen in Figure 3, the explants treated with serum-free medium (negative control) remained intact. In contrast, treatment of the explants with a specific Ca2+ ion chelator, EGTA (positive control), significantly increased the intercellular space in the respiratory epithelium (*p* < 0.05) by disrupting the ICJ between the epithelial cells (Figure 3A). The explants treated with spore diffusate at a high dilution (1:250) showed a significantly higher percentage of intercellular space (*p* < 0.05) in the epithelium than the control explants (Figure 3B). At a low dilution (1:25), the spore diffusate treatment induced desquamation of columnar epithelial cells and, thus, exposure of the basal cell layer. The spore diffusate treatment at dilution 1:250 did not significantly decrease the epithelial thickness compared to SFM or EGTA treatment (Figure 3C).

### 3.4. Spore Proteases Predispose the Respiratory Epithelium for EHV-1 Infection 

**Figure 4:**
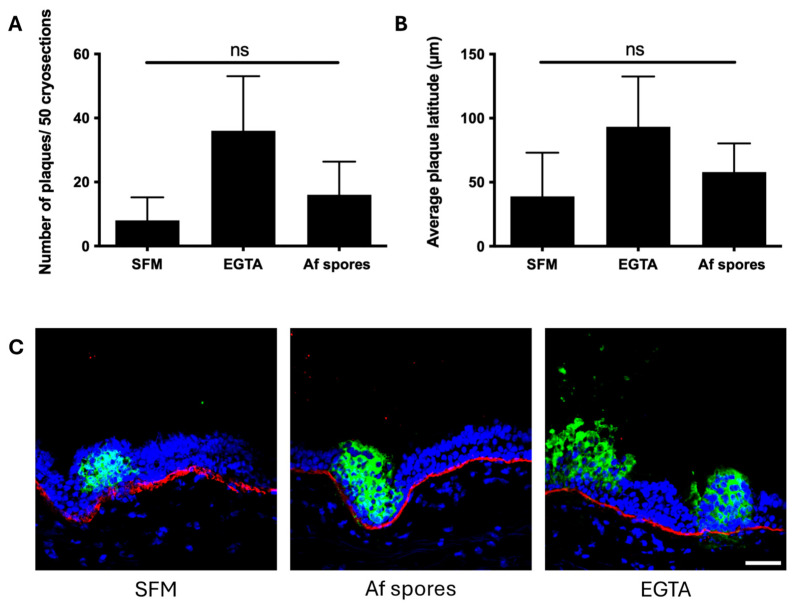
EHV-1 infection of the respiratory epithelium after spore diffusate treatment. (**A**) Explants were treated with SFM (negative control), EGTA treatment (positive control), or the spore diffusate of the clinical isolate *Aspergillus fumigatus* at a dilution of 1:250 for 24 h. Cryosections were made and stained for EHV-1 late glycoproteins to assess the EHV-1 infection by counting the number of new plaques in 50 consequent cryosections. (**B**) The maximum plaque latitude of each new EHV-1 plaque was also determined. Data are represented as a mean of three replicates with standard deviation. No statistical significance was observed, indicated by non-significance (ns). (**C**) Representative confocal images of EHV-1 plaques for the different pretreated mucosal explants are shown. EHV-1 late glycoprotein was stained in green, basement membrane in red, and nuclei in blue. Scale bar measures 100 µm.

We next investigated whether the disruption of the epithelial ICJ would make the respiratory epithelium more susceptible to EHV-1 infection. As seen in Figure 4A, the number of plaques per 50 cryosections increased from 8 ± 7 in SFM-pretreated explants (negative control) to 16 ± 10 upon spore diffusate pretreatment. In the positive control, the number of plaques increased to 36 ± 17 after EGTA pretreatment (*p =* 0.0704). No significant differences were found in the number of plaques for the different treatments. The plaque latitude was also measured, which gives an indication of viral spread efficiency in the respiratory epithelium. In Figure 4B, we showed that the average EHV-1 plaque latitude increased from 39 ± 32 µm in SFM pretreated explants to 58 ± 22 µm in spore diffusate pretreatment. Similarly, the average plaque latitude increased to 93 ± 39 µm after EGTA treatment. Again, no significant differences were found in the average plaque latitude for the different treatments. Representative confocal images of the EHV-1 plaques for the different pretreated mucosal explants are shown in Figure 4C.

## 4. Discussion

The findings of this study highlight the critical impact of environmental air contaminants, particularly *Aspergillus fumigatus* (Af) spores, on equine respiratory health. Horses are vulnerable to airborne contaminants due to their extensive time spent in enclosed environments. Stables and barns can concentrate harmful Af spores due to dust accumulation and hay as a source of high spore concentrations [49,50]. The respiratory epithelium functions as a barrier in a multifaceted defense system against airborne contaminants. The mucus barrier is an important site of conidia-host interactions since most of the Af conidia are removed in healthy individuals by mucus entrapment and mucociliary transport [17]. By secreting proteases and glycosidases, Af conidia may penetrate the mucus after degrading mucin proteins and carbohydrates [51]. *A. fumigatus* conidia mainly adhere to mucus, microscopic indentations of damaged epithelial cells, and, directly, epithelial cells that are interconnected by ICJ [47]. It was demonstrated that Af conidia adhere to and invade bronchial epithelial cells, leading to the inhibition of ciliary beating and disruption of the epithelial integrity with cell detachment [52,53]. This destruction of epithelial integrity was previously attributed to Af proteases [22]. One of the ICJ targets of spore proteases was the tight junctions in the respiratory epithelium [54]. 

The destruction of ICJ predisposes the equine respiratory epithelium to respiratory viral infections such as EHV-1. When the epithelial junctions are compromised, the basolateral receptor of EHV-1 becomes freely accessible to the virus [37]. It was recently demonstrated that pollen proteases selectively and irreversibly disrupted these epithelial junctions and, thus, increased viral infection by EHV-1 [40]. Furthermore, we hypothesized that Af spore proteases similarly induce loss of epithelial barrier function, which may facilitate the invasion of the alphaherpesvirus EHV-1. To our knowledge, we are the first to investigate the effect of Af spore diffusate on an ex vivo tracheal explant system. An in-house developed equine respiratory mucosa explant system was used to investigate the hypothesis. Although this system will never be a strict copy of the in vivo situations and processes, it has been demonstrated to be a reliable model for mimicking in vivo conditions, whereby the respiratory mucosa explants maintain viability (>90%) after culturing and a following positive control treatment with EGTA [37,43]. 

Morphological analysis of the tracheal respiratory epithelium, based on HE staining of tissue sections, revealed that the integrity of the epithelium was drastically altered by the Af spore diffusate. Consistent with previous studies, the spore diffusate induced desquamation of the epithelial cells and a great significant increase of intercellular space at, respectively, high and low concentrations [22,55]. The disruption of the epithelial integrity may be a direct consequence of proteolytic actions or may derive indirectly from the activation of the protease-activated receptor (PAR) [56]. Af has been shown to activate PAR-2 in bronchial epithelial cells, which consequently enhanced the expression of PTPN11, a phosphatase known to inhibit IFN signaling, and suppressed the expression of CXCL10 mRNA, a Th1 chemokine. This action of Af may promote viral infections and promote Th2 inflammation responses in allergic airway disorders, mediated by Af [57]. We have demonstrated that the epithelium was more easily infected by EHV-1 upon ICJ disruption by Af spore proteases. An increase in EHV-1 plaques, as well as an increase in plaque latitude, was observed. These results align with those of a previous study using pollen diffusate, in which similar effects caused by pollen proteases were demonstrated. [40]. Although the increase in infection upon Af spore diffusate pretreatment was not as pronounced as upon pollen protease pretreatment, a clear and noticeable trend of increased infection by EHV-1 was evident. 

By means of mass spectrometry (MS)-based proteomics, we have identified 11 different proteases in the Af spore diffusate belonging to the serine protease, metalloprotease, and aspartic protease groups. The presence of a large number of serine proteases (7) supports a potential contribution of PAR-2 since it is selectively activated by serine proteases through the cleavage of its N-terminus [58]. Proteases of Af are involved in signaling, metabolic regulations, tissue degradation, and nutrient acquisition. Some of these proteases have been shown to be virulence factors of Af [59]. 

The serine protease group is the biggest group represented in the spore diffusate, with seven different identified serine proteases. The identified serine proteases alkaline protease 1 and 2 (Alp1 and 2) belong to the subtilisin family and can degrade components of the extracellular matrix (ECM) by collagenolytic and elastase activity [60]. Alp1 in particular has been shown to be a virulence factor and allergen with the ability to invade the respiratory mucosa by degrading ECM [61]. Furthermore, Alp1 helps the Af conidia to evade the host immune response by evading opsonization [62]. The serine proteases dipeptidyl-peptidase 4 and 5 (DppIV and DppV) were suggested to play a role in the digestion of invaded tissues and modulation or induction of the immune response upon conidia inhalation [63]. The last three serine proteases, Carboxypeptidase Y homolog A, Tripeptidyl-peptidase sed3, and Proline iminopeptidase, which were identified in the Af spore diffusate, are unknown to be of pathogenic importance.

Next, we have identified three metalloproteases in the Af spore diffusate, including zinc metallopeptidase, vacuolar aspartyl aminopeptidase Lap4, and Xaa-Pro aminopeptidase pepP. The metalloprotease group has been shown to degrade collagen and elastin, contributing to tissue destruction and invasion. Compared to serine proteases, their proteolytic activity is less extensive [64].

The last identified protease group is the aspartic protease group, in which we identified beta-aspartyl-peptidase. It was suggested that this protease group may solely play a role in fungal morphogenesis [14].

Besides proteases, a range of glycosidases were identified, as was one lipase. The glycosidases of Af have been shown to degrade the mucus barrier on the respiratory epithelium together with aspartyl and subtilisin proteases by degrading mucin carbohydrates and mucin proteins, respectively [65]. The identified lipase, named phosphatidylglycerol specific phospholipase, degrades the anionic lipid phosphatidylglycerol, which is the most abundant lipid in lung surfactant and is present in low amounts in other mammalian membranes [66].

The destruction of intercellular junctions due to pollen proteases was primarily caused by serine proteases of the subtilisin family [40]. In our study, an arsenal of proteases in the Af spore diffusate were identified, including the serine proteases Alp1 and Alp2 from the subtilisin family. Alp1 has been shown to damage tight junctions and E-cadherins, which resulted in a compromised barrier integrity and reduced transepithelial electrical resistance (TEER) [67]. This may indicate that the destruction of intercellular integrity is mainly carried out by serine proteases, particularly proteases of the subtilisin family. Further investigation into the role of this protease family in other respiratory pathogens or allergens could be interesting. In comparison with the study using pollen diffusate pretreatment, the increase in infection by EHV-1 following treatment with spore diffusate was not statistically significant. Additionally, many more protease groups and glycosidases were identified in the Af spore diffusate compared to the pollen diffusate [40]. We hypothesize that the mechanism underlying this lack of significance might involve the cleavage and inactivation of the viral receptor through proteolytic or glycosidase activity. Efficient EHV-1 entry, as with other herpesviruses, is dependent on the interaction with cell surface glycosaminoglycans (GAG) [68]. We have demonstrated that the Af spore diffusate includes 35 different glycosidases that can interact with glycosaminoglycans at the cell surface and possibly alter their function. The deglycosylation of the GAG may result in a decrease in viral binding and, subsequently, entry into host cells. This hypothesis is supported by findings that the alphaherpesvirus herpes simplex virus type 1 (HSV-1) expresses glycosidase activity to release progeny viruses from the receptors and to inhibit the re-entry of parent cells. A similar effect is expected from proteases, as HSV-1 binds a GAG known as heparan sulfate (HS) proteoglycan, which can be degraded through the cleavage of HS and the associated proteoglycan [69].

Furthermore, Af proteases have been shown to disrupt airway epithelial cells, as previously discussed, consequently inducing pro-inflammatory cytokines and driving mucus production. Excessive mucus production can facilitate the accumulation and growth of inhaled conidia, leading to prolonged secretion of proteolytically active allergens. This process initiates a cycle of injury and repair, resulting in the loss of epithelial integrity and long-term structural changes in the airways [70]. A concurrent enhanced replication of (alphaherpes)viruses in the respiratory epithelium may exacerbate epithelial cell damage, leading to greater mucus accumulation and the disruption of mucociliary clearance [71]. Furthermore, the epithelial damage will facilitate the continuous release of antigens and allergens to trigger a robust activation of the Th2-type immune response, leading to an increase in both total and specific IgE antibodies [72]. This may lead to allergic hypersensitivity diseases of the airways such as Severe Asthma with Fungal Sensitization (SAFS) and Allergic Bronchopulmonary Aspergillosis (ABPA) [73]. Recently, the development of an allergic response was demonstrated in mice after repeated exposure to Af conidia [74]. Severe equine asthma, also known as recurrent airway obstruction, is a common chronic respiratory disease with a prevalence of 14% in a horse population measured in Great Britain [75]. Concurrent respiratory viral infections may also contribute to the development of asthma, with herpesviruses being the most abundant asthma-associated viruses [76].

## 5. Conclusions

Our research provides valuable insights into the mechanisms by which *Aspergillus fumigatus* (Af) spores compromise equine respiratory health and, consequently, have a role in respiratory viral infections. More precisely, we have demonstrated that Af spores significantly compromise equine respiratory epithelial integrity through proteolytic activity. The compromised epithelium, characterized by epithelial damage and increased intercellular space, showed an increased susceptibility of the respiratory epithelium to EHV-1 infections upon exposure to Af spore proteases. We have identified many different proteases by means of mass spectrometry (MS)-based proteomics, belonging to the serine protease, metalloprotease, and aspartic protease groups. Furthermore, we propose that concurrent EHV-1 replication may exacerbate allergic responses to Af spores through mechanisms yet to be elucidated. These findings highlight the need for good management practices to reduce Af spore exposure and protect equine respiratory health.

## Figures and Tables

**Table 1:** List of proteases in the clinical Af spore diffusate. These enzymes were identified via mass spectrometry-based proteome analysis of three regions (100–250 (A); 60–90 (B); 30–50 (C)) in an SDS-PAGE of *aspergillus fumigatus* clinical isolate spore diffusate, corresponding to the proteolytic activity in the zymography. A full list of the proteases, glycosidases, and lipases in the Af spore diffusate is given in the Supplementary Materials Table S1.

Type	UniprotKB Protein Accession Number	Description	Corresponding Af Gene Annotation	Lane (kDa)	Sequence Coverage %	Nr. of Unique Peptides
Serine protease	P28296	Alkaline protease 1	AFUA_4G11800	100–250 60–90 30–50	18.6 21.1 5.5	7 8 3
Serine protease	P87184	Alkaline protease 2	AFUA_5G09210	30–50	8.9	4
Serine protease	P0C959	Dipeptidyl-peptidase 5	AFUA_2G09030	100–250 60–90 30–50	31.9 3.2 12.1	5 2 7
Serine protease, aminopeptidase	Q4WPH9	Probable dipeptidyl peptidase 4	AFUA_4G09320	100–250	7.2	4
Serine protease	Q5VJG9	Carboxypeptidase Y homolog A	AFUA_6G13540	30–50	6.4	3
Serine protease	Q70GH4	Tripeptidyl-peptidase sed3	AFUA_3G08930	30–50	2.9	1
Serine protease, aminopeptidase	Q4WHL2	Proline iminopeptidase	AFUA_2G05000	100–250 30–50	20.3 2.2	9 1
Metalloprotease	Q4WMK3	Zinc metallopeptidase, putative	AFUA_6G09600	30–50	5.2	2
Metalloprotease, aminopeptidase	Q4WES0	Vacuolar aspartyl aminopeptidase Lap4, putative	AFUA_5G03990	100–250 30–50	21.7 12.4	11 5
Metalloprotease, aminopeptidase	Q4X267	Probable Xaa-Pro aminopeptidase pepP	AFUA_2G07500	30–50	11.8	1
Aspartic protease	Q4WHT3	beta-aspartyl-peptidase	AFUA_2G04280	60–90	3.9	1

## Data Availability

Raw data are available from the corresponding author upon reasonable request.

## Supplementary Materials

The following supporting information can be downloaded at: https://www.mdpi.com/article/10.3390/v16081208/s1, Figure S1: Cell viability in respiratory mucosal explants; Table S1: List of proteases, glycosidases and lipases in the clinical spore diffusate.
