# Supplementary material for: Aspergillus Fumigatus Spore Proteases Alter the Respiratory Mucosa Architecture and Facilitate Equine Herpesvirus 1 Infection
Source: Viruses. 2024 Jul 27;16(8):1208. doi: 10.3390/v16081208 (PMC11358968; doi:10.3390/v16081208)
Supplement: Supplementary file 1 [file viruses-16-01208-s001.zip › viruses-3106534-supplementary.pdf]

Supplementary information

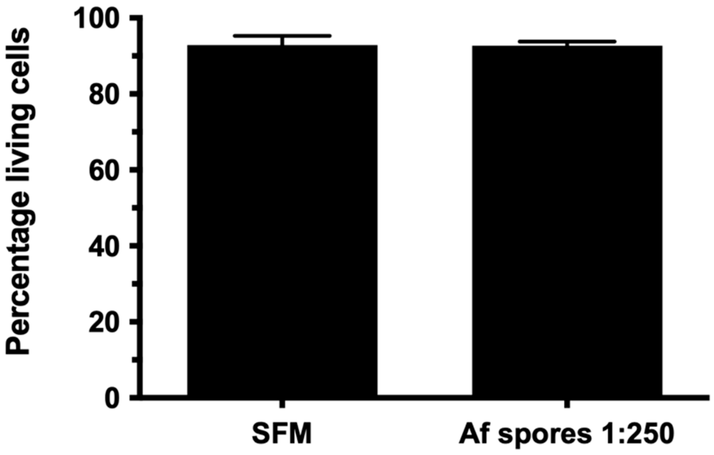

Figure S1. Cell viability in respiratory mucosal explants. The spore diffusate treatment does not reduce the viability of the respiratory mucosal explants. The percentage of living cells was measured by counting the apoptotic cells of 3 replicates in 3 different randomly chosen regions in the epithelium. The percentage of living cells after treatment with Aspergillus fumigatus clinical isolate 3414 spore diffusate was determined and compared to the SFM treatment (negative control).

Table S1. List of proteases, glycosidases and lipases in the clinical spore diffusate.

Type	UniprotKB protein accession number	Description	Corresponding Af gene annotation	Lane (kDa)	Sequence coverage %	Nr. of unique peptides
Serine protease	P28296	Alkaline protease 1	AFUA_4G11800	100-250<br>60-90	18.6<br>21.1	7<br>8
Serine protease	P87184	Alkaline protease 2	AFUA_5G09210	30-50	8.9	4
Serine protease	P0C959	Dipeptidyl-peptidase 5	AFUA_2G09030	100-250<br>60-90	31.9<br>3.2	5<br>2
Serine protease, aminopeptidase	Q4WPH9	Probable dipeptidyl peptidase 4	AFUA_4G09320	100-250	7.2	4
Serine protease	Q5VJG9	Carboxypeptidase Y homolog A	AFUA_6G13540	30-50	6.4	3
Serine protease	Q70GH4	Tripeptidyl-peptidase sed3	AFUA_3G08930	30-50	2.9	1
Serine protease, aminopeptidase	Q4WHL2	Proline iminopeptidase	AFUA_2G05000	100-250<br>30-50	20.3<br>2.2	9<br>1
Metalloprotease	Q4WMK3	Zinc metallopeptidase, putative	AFUA_6G09600	30-50	5.2	2
Metalloprotease, aminopeptidase	Q4WES0	Vacuolar aspartyl aminopeptidase Lap4, putative	AFUA_5G03990	100-250<br>30-50	21.7<br>12.4	11<br>5
Metalloprotease, aminopeptidase	Q4X267	Probable Xaa-Pro aminopeptidase pepP	AFUA_2G07500	30-50	11.8	1
Aspartic protease	Q4WHT3	beta-aspartyl-peptidase	AFUA_2G04280	60-90	3.9	1
Glycosidase	Q4WJJ3	Probable beta-glucosidase A	AFUA_1G05770289	100-250<br>60-90	24.9<br>1.9	5<br>1
Glycosidase	Q4W962	Glycosyl hydrolase, putative	AFUA_4G00390	100-250	16.5	15

Glycosidase	Q4WT26	Alpha-1,2-mannosidase family protein, putative	AFUA_1G10790	100-250	21.6	16
Glycosidase	Q4WZ74	Glycoside hydrolase family 43 protein	AFUA_2G17920	100-250	14.0	10
Glycosidase	Q4WKA6	Glycosyl hydrolase, putative	AFUA_1G03140	100-250<br>30-50	15.9<br>1.8	8<br>1
Glycosidase	Q4WHW3	Alpha-1,3-glucanase/mutanase, putative	AFUA_2G03980	100-250<br>60-90	5.6<br>13.5	2<br>6
Glycosidase	Q4X068	Hydrolase, putative	AFUA_2G14520	100-250	2.5	1
Glycosidase	Q4WHH3	Sugar hydrolase, putative	AFUA_2G05400	100-250	1.6	1
Glycosidase	Q4WX47	alpha-mannosidase	AFUA_3G08200	100-250	33.3	45
Glycosidase	Q4WCB5	Beta-hexosaminidase	AFUA_8G05020	100-250	25.3	14
Glycosidase	Q4WVS8	Agmatinase, putative	AFUA_5G13180	100-250	4.3	2
Glycosidase	Q4WRH9	Probable alpha/beta-glucosidase agdC	AFUA_1G16250	100-250	15.6	13
Glycosidase	Q4WR62	Probable beta-glucosidase M	AFUA_1G17410	100-250	9.4	6
Glycosidase	Q4WMU3	Probable beta-glucosidase F	AFUA_6G08700	100-250	12.1	8
Glycosidase	Q4WTB3	Probable alpha-L-arabinofuranosidase C	AFUA_1G09900	100-250	4.4	2
Glycosidase	Q4WPR1	alpha-glucosidase	AFUA_4G10150	100-250	4.0	3
Glycosidase	Q4WIT7	Glucoamylase	AFUA_2G00690	100-250<br>60-90	2.7<br>15.8	2<br>9
Glycosidase	E9R226	Exo-beta-1,3-glucanase Exg0	AFUA_1G14450	60-90<br>30-50	24.0<br>9.2	21<br>4
Glycosidase	Q4WRZ5	Probable mannosyl-oligosaccharide alpha-1,2-manno-	AFUA_1G14560	60-90<br>30-50	39.6<br>23.3	21<br>11
Glycosidase	Q4WWY2	Exo-beta-1,3-glucanase, putative	AFUA_3G07520	60-90<br>30-50	27.3<br>13.7	19<br>9
Glycosidase	Q4WJZ7	glucan endo-1,3-beta-D-glucosidase	AFUA_1G04260	60-90<br>30-50	22.9<br>2.2	17<br>1
Glycosidase	Q4WLJ9	Exo-beta-1,3-glucanase, putative	AFUA_6G13270	60-90<br>30-50	20.5<br>5.6	10<br>3
Glycosidase	Q4WGT3	Probable beta-glucosidase L	AFUA_7G06140	60-90	16.4	11
Glycosidase	Q4WV22	Alpha-1,2-mannosidase family protein	AFUA_5G10520	60-90	3.8	2
Glycosidase	Q4WG16	Probable glucan endo-1,3-beta-glucosidase eglC	AFUA_3G00270	60-90<br>30-50	6.3<br>20.6	3<br>8
Glycosidase	Q4WIU2	beta-N-acetylhexosaminidase	AFUA_2G00640	60-90	2.4	2
Glycosidase	Q4WIR3	Xylosidase/glycosyl hydrolase, putative	AFUA_2G00930	60-90	2.3	1

Glycosidase	Q4W930	Probable arabinan endo-1,5-alpha-L-arabinosidase C	AFUA_6G00770	30-50	18.7	6
Glycosidase	Q4WHR4	Xylosidase/arabinosidase, putative	AFUA_2G04480	30-50	21.0	5
Glycosidase	Q4WQR8	Glycosyl hydrolase, putative	AFUA_4G13770	30-50	26.3	6
Glycosidase	Q4WI46	Probable glycosidase crf2	AFUA_2G03120	30-50	7.9	3
Glycosidase	Q4WF34	xyloglucan-specific endo-beta-1,4-glucanase	AFUA_3G03610	30-50	11.1	3
Glycosidase	Q4WRY0	Beta-glucosidase, putative	AFUA_1G14710	30-50	4.1	1
Glycosidase	Q4WMS9	Beta-mannosidase A	AFUA_6G08840	30-50	1.5	1
Glycosidase	Q4WE86	Probable alpha-galactosidase B	AFUA_5G02130	30-50	2.5	1
Lipase	Q4WFP1	Phosphatidylglycerol specific phospholipase, putative	AFUA_3G01530	30-50	10.4	4

These enzymes were identified via mass spectrometry-based proteome analysis of three regions (100-250 (A); 60-90 (B); 30-50 (C)) in an SDS-PAGE of aspergillus fumigatus clinical isolate spore diffusate, corresponding to the proteolytic activity in the zymography.
